# The Impact of Alcohol Consumption Pattern on Liver Fibrosis in Asymptomatic Patients

**DOI:** 10.3390/jcm12237405

**Published:** 2023-11-29

**Authors:** Horia Minea, Ana-Maria Singeap, Catalin Victor Sfarti, Irina Girleanu, Stefan Chiriac, Cristina Muzica, Tudor Cuciureanu, Oana Cristina Petrea, Laura Huiban, Sebastian Zenovia, Robert Nastasa, Adrian Rotaru, Remus Stafie, Ermina Stratina, Camelia Cojocariu, Carol Stanciu, Anca Trifan

**Affiliations:** 1 Department of Gastroenterology, Grigore T. Popa University of Medicine and Pharmacy, 700115 Iasi, Romania; horia.minea@yahoo.com (H.M.); lungu.christina@yahoo.com (C.M.); drcuciureanutudor@gmail.com (T.C.); huiban.laura@yahoo.com (L.H.); sebastianzenovia20@gmail.com (S.Z.); robertnastasa948@gmail.com (R.N.); adrianrotaru94@yahoo.com (A.R.); stafieremus@gmail.com (R.S.); stratina.ermina@yahoo.com (E.S.); cameliacojocariu@yahoo.com (C.C.); stanciucarol@yahoo.com (C.S.); ancatrifan@yahoo.com (A.T.); 2 Institute of Gastroenterology and Hepatology, “St. Spiridon” University Hospital, 700111 Iasi, Romania

**Keywords:** alcohol consumption, advanced fibrosis, screening, transient elastography

## Abstract

Introduction: Alcohol consumption (AC) represents a widespread cause of liver diseases affecting 10–20% of the population. The study aimed to evaluate the relationship between advanced liver fibrosis (ALF) measured by transient elastography (TE), laboratory parameters, and the amount of AC depending on non-modifiable risk factors such as age and gender. Methods: We examined 689 patients with an average age of 49.32 ± 14.31 years, 72.9% males, without liver pathology, who admitted a moderate/high consumption (female ≤ 7 versus > 7 drinks/week; male ≤ 14 versus > 14 drinks/week) for at least five years. The fibrosis level was adjusted according to transaminase levels. Predictive factors were established using univariate regression analysis. Results: ALF (≥F3) was detected in 19.30% of subjects, predominantly males (14.1%) and patients over 55 years (12.5%). Excessive consumption of distilled spirits is associated with ALF in females (OR = 4.5), males (OR = 6.43) and patients over 55 years (OR = 3.73). A particularity highlighted in both genders, regardless of the age group, was the negative correlation between the decrease in the number of platelets, the albumin concentration, and the appearance of AFL. Conclusions: Screening using TE represents an approach for early detection of ALF in asymptomatic populations and the development of a risk stratification scheme.

## 1. Introduction

Excessive alcohol consumption, as part of an unhealthy lifestyle, has detrimental consequences for personal and social development, as well as health. It represents a risk factor linked to over 200 diseases [1,2]. Globally, it stands as the third leading cause of premature mortality, contributing to over 3,000,000 deaths recorded annually, predominantly among individuals aged 15 to 49 [3,4]. Currently, it is estimated that 5.1% of the global burden of diseases and injuries (DALY) is attributed to this cause [3,5].

According to data reported by the World Health Organization, Europe stands out with a high alcohol consumption rate (an average of 10.7 L annually). At least one episode of abuse per week (defined as ≥5 drinks on an occasion) is reported for one-fifth of individuals aged 15 and above [4,6,7].

With a rate of 35%, significantly surpassing the European Union average of 20%, Romania ranks second in reporting episodes of excessive alcohol consumption per month. This trend is more pronounced among males, with a prevalence of 26.3% compared to 11.4% in females [8]. Alcohol-induced liver injury manifests across a broad spectrum of presentations, progressing gradually toward fibrosis, cirrhosis, and hepatocellular carcinoma [9,10].

An analysis of morbidity data recorded in 14 countries from the European Union has shown that a 1-L increase in alcohol consumption per capita led to a 10% increase in the incidence of cirrhosis [6,11]. With a continuously rising prevalence of liver cirrhosis (increasing from 96.3/100,000 inhabitants in 1990 to 118.3/100,000 population in 2017) and ranking first worldwide in new cases of liver cancer (2.6/100,000 inhabitants), alcohol consumption poses a serious threat to health in Romania [12,13].

The type of beverages, quantity, and pattern of alcohol consumption in relation to age, gender, and clinical and paraclinical factors partially explain individual differences in susceptibility to the development of hepatic fibrosis [14,15]. To prevent the risk of complications due to cirrhosis, an early diagnosis of hepatic fibrosis could influence the patient’s decision to limit and even cease alcohol consumption. Although considered the gold standard for fibrosis assessment, the liver biopsy approach has witnessed reduced patient compliance due to its invasive nature, which could lead to the occurrence of serious complications and increased mortality [16]. Moreover, the high costs and the influence on the accuracy of histological examination through sampling errors and interobserver interpretation variability limit the use of this procedure in medical practice [17].

In this context, our objective was to examine the prevalence of advanced liver fibrosis, assessed through transient elastography, in the asymptomatic population with alcohol consumption. We aimed to evaluate the pattern of alcohol consumption in relation to non-modifiable factors (such as age and gender), clinical parameters, and laboratory results to establish predictive models.

## 2. Materials and Methods

### 2.1. Patients

**Figure 1:**
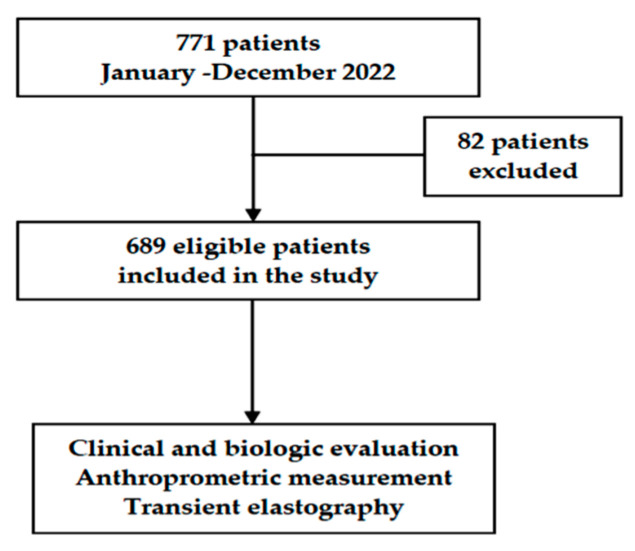
Flow chart of patient selection.

Between January and December 2022, we conducted a cross-sectional study at a gastroenterology and hepatology outpatient clinic within a tertiary referral hospital situated in the Northeastern region of Romania. The patients presented for consultations motivated by various complaints in the gastroenterological sphere (excluding emergency acute settings) or for routine check-ups. The inclusion criteria were individuals aged 18 years or older who signed the informed consent form, with no prior history of chronic liver disease, and a self-reported history of alcohol consumption for a minimum of the past 5 years. Exclusion criteria were a history of known chronic disease, overt cirrhosis, identification of other causes of liver disease, non-drinkers (defined as individuals who abstain from alcohol entirely), and pregnant women. Demographic information, along with clinical, paraclinical, and imaging findings, were obtained from the patients’ electronic health records. A total of 771 patients were examined; 689 subjects were eligible for the study, and 82 were excluded. Figure 1).

The data regarding the consumption pattern were based on self-reporting, the interview questions covering the amount expressed in drinks units, frequency, and type of preferred drinks (wine, beer, or distilled spirits). According to the Dietary Guidelines for Americans, we defined one standard drink as 14 g of ‘‘pure” alcohol being the equivalent of a 330 mL bottle of beer with about 5% alcohol, 150 mL glass of wine with about 12% alcohol, or 50 mL distilled spirits, with about 40% alcohol. This information was used to divide the study participants into two categories: moderate consumption (women < 7 drinks/week; men < 14 drinks/week) and excessive consumption (≥7 drinks per week for women or ≥14 drinks per week for men) [18,19].

### 2.2. Vibration Controlled Transient Elastography

The evaluation of liver stiffness was performed for all the patients using Vibration-Controlled Transient Elastography (VCTE) (Echosens, Paris, France). The M or XL probes were chosen using the device’s automatic probe selection tool software. All the participants were examined under fasting conditions for a minimum of four hours while lying down with the right hand above the head and focusing on the right lobe of the liver through an intercostal space in a region without major vessels [20].

The examination was regarded as valid if 10 measurements were performed with a mean interquartile range below 30%. The results of the liver stiffness were expressed in kilopascals using the following cut-offs to establish the stages of the fibrosis: <4.9 kPa for the absence of fibrosis (F0), mild to moderate fibrosis (F1–F2) between ≥4.9 kPa and <8.1 kPa, advanced fibrosis (F3) ≥ 8.1 kPa and <10.5 kPa, and ≥10.5 kPa for liver cirrhosis (F4). Moreover, the classification of the fibrosis stage was adjusted in patients with elevated values of the hepatic transaminases according to the formulas developed by Mueller et al.: F_0_ < 4.9 ^exp 0.0022 x AST^; F_1_–F_2_ ≥ 4.9 ^exp 0.0022 x AST^ and <8.1^exp 0.0046 x AST^; F_3_ ≥ 8.1^exp 0.0046 x AST^ and < 10.5 ^exp 0.0069 x AST^; F_4_ ≥ 10.5 ^exp 0.0069 x AST^) [21].

### 2.3. Statistical Analysis

Measured values and data dispersion were expressed as means, medians, interquartile range, and confidence intervals. The Kolmogorov–Smirnov test was used to evaluate the normal distribution of the numerical variables. The differences between the groups of patients with liver fibrosis ≤ F2 and ≥F3 were evaluated using the *t*-test for continuous variables. At the same time, the Kruskal–Wallis test was used to examine the differences between laboratory parameters according to the type of drink consumed and gender of the patient in those with advanced fibrosis.

The association of hypothetical predictors with liver stiffness was evaluated using the Pearson correlation coefficient. The SPSS program (Inc., Chicago, IL, USA, version 20) was used, and the level of significance chosen for statistical tests was *p* < 0.05.

## 3. Results

Demographic characteristics and clinical data of the 689 patients included in the study are described in Table 1. Liver stiffness compatible with advanced fibrosis (≥F3) was predominantly detected in male patients and in the age group over 55 years, being associated with increased alcohol consumption.

In Table 2, the biomarkers used for assessing liver function, inflammation, and lipid status of the subjects included in the study are presented in relation to the pattern of alcohol consumption (excessive versus moderate). Comparative analysis revealed significant increases in liver enzymes (ALT, AST, GGT, ALP), total bilirubin, and triglycerides in individuals who reported excessive alcohol consumption.

**Figure 2:**
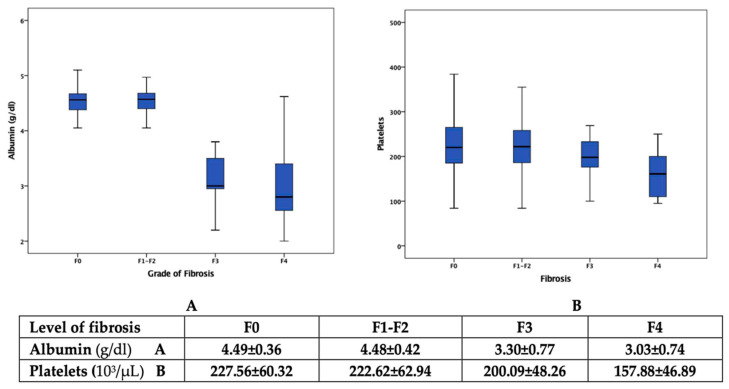
Distribution of the albumin and platelets according to the level of fibrosis.

Moreover, the abnormal values of albumin and the number of platelets are noted, with a decrease in these biomarkers proportional to the increase in the degree of liver fibrosis (Figure 2).

Table 3 contains data regarding the variation of biomarker concentration in patients with advanced fibrosis (≥F3) according to gender and the preferred type of alcoholic beverage. It was observed that male subjects who consume alcoholic drinks have significant changes in liver enzymes (AST, ALT), total cholesterol, LDL-cholesterol, triglycerides, and CRP. Instead, the same beverage influenced only the lipid profile and CRP in the case of female subjects. Regardless of the type of drinks consumed, in both genders, a low level of HDL cholesterol and albumin was found. Due to the reduced number (n = 2), females diagnosed with advanced fibrosis associated with beer consumption were excluded from the analysis.

**Figure 3:**
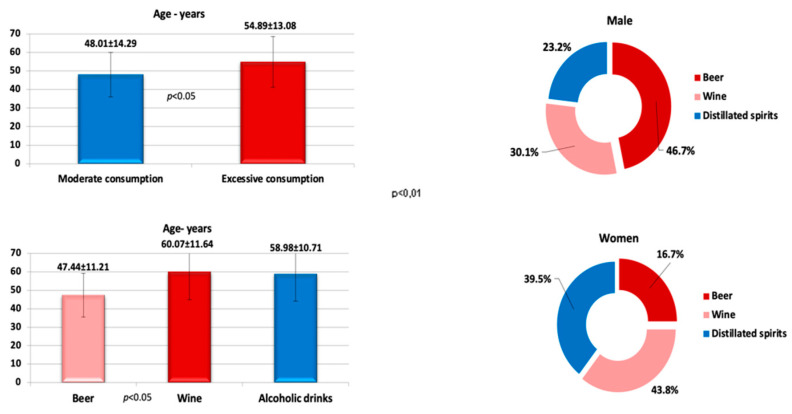
The pattern of alcohol consumption according to age and gender.

Excessive consumption was associated with people with an average age of 54.89 ± 13.08 years who prefer spirits and wine, while moderate consumption, based mainly on beer, was frequent among those under the age of 50 (*p* < 0.01). Considering the distribution based on the gender of the patients included in the study, it is noticed that beer consumption was predominant in males, while females preferred wine and distilled spirits in comparable proportions (Figure 3).

**Figure 4:**
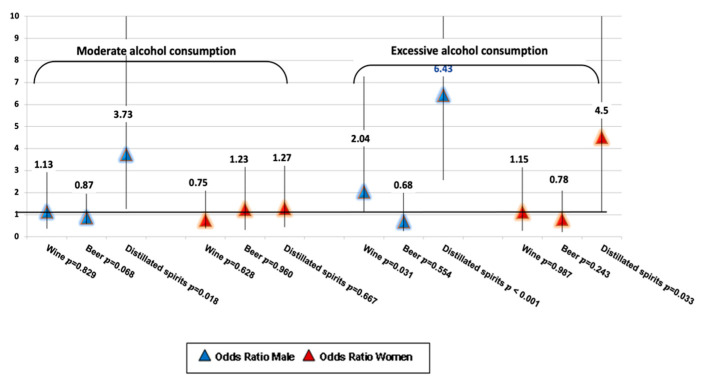
Risk of advanced fibrosis according to the pattern of alcohol consumption.

The risk for advanced fibrosis due to excessive consumption was more than three times higher for distilled spirits (OR = 6.43, 95% CI: 1.67–10.43) compared to wine (OR = 2.04, 95% CI: 1.12–4.51) in men. Moreover, there was a significant correlation between advanced fibrosis and alcoholic beverages for moderate consumption in men (OR = 3.73, 95% CI: 1.35–7.87), respectively, excessive alcohol consumption in women (OR = 4.5, 95% CI: 3.29–9.50) (Figure 4).

In patients over 55 years old, only distilled spirits, regardless of the type of consumption (moderate or high), present comparable risk values (OR = 3.62, 95% CI: 1.13–11.64 versus OR = 3.73, 95% CI: 1.51–14.63) for the occurrence of advanced fibrosis according to the results in Table 4.

Table 5 presents the results of the univariate analysis to identify independent predictors associated with advanced fibrosis. The gender-dependent variation shows a substantial correlation between the presence of liver stiffness, triglycerides (β = 0.343, *p* = 0.036), and total bilirubin (β = 0.257, *p* < 0.001) in male patients, respectively, liver enzymes (GGT β = 0.458, *p* < 0.001, ALT β = 0.240, *p* < 0.001; AST β = 0.212, *p* < 0.001) in females. After the age of 55, the strongest associations were observed for LDL-c (β = 0.384, *p* < 0.001), total bilirubin (β = 0.314, *p* < 0.001), and liver enzymes (ALT β = 0.278, *p* < 0.001; AST β = 0.290, *p* < 0.001). A particularity highlighted in both genders, regardless of the age group, was the establishment of a negative correlation between the decrease in the number of platelets (*p* < 0.001), the albumin concentration (*p* < 0.001), and the appearance of advanced fibrosis. The other clinical and laboratory parameters included in Table 1 and Table 2 had no statistical significance for the univariate analysis (Table 5).

## 4. Discussion

Even though significant morbidity and mortality due to alcohol abuse are annually reported worldwide, the relationship between the amount, patterns of consumption, preferred beverage, and the impact on health is not clearly defined [14,15]. This data is regarded as the reason for the conceptualization of this study, whose results reflected the aim: almost 20% of the included subjects were diagnosed with advanced fibrosis and were at risk of decompensation due to liver disease.

Alcohol induces cell damage with the release of endotoxin, which influences both natural and adaptive immunity. Numerous cytokines and chemokines are synthesized, with a stimulatory effect on the proliferation of T or B lymphocytes, which also activate collagen-producing stellate cells. Due to the complex interaction, signs of chronic inflammation are developed with the liver parenchyma being replaced by non-functional connective tissue, a process that progressively evolves toward cirrhosis in a considerable number of patients [22,23]. Microbial dysbiosis generated by the action of oxidative and non-oxidative metabolites of ethanol affects the integrity of the intestinal barrier and disrupts permeability, which stimulates the progression of inflammation, the appearance of fibrosis, and the development of alcoholic liver disease (ALD) [24,25,26].

The incidence of liver diseases associated with this etiology is higher in men, but the risk of disease is much more increased in women, a susceptibility that could be explained by the lower volume of alcohol distribution, which leads to a significant concentration in the blood. Additionally, the stimulating influence of estrogen hormones on intestinal permeability and the regulation of endotoxin receptors in Kupffer cells is added, with the release of significant amounts of tumor necrosis factor [27].

The protective effect on health associated with low amounts of alcohol has been frequently mentioned in the medical literature, without there being any evidence to establish an optimal dose for daily or weekly consumption [28]. It is noted that, above all, red wine provides significant benefits compared to other alcoholic beverages, probably due to the presence of bisphenols that decrease blood pressure, inhibit the oxidation of low-density lipoproteins, and have a favorable effect on the cellular redox state, reducing platelet aggregation and inflammatory processes [29,30].

Significant arguments against this concept were reported in the Global Burden of Diseases, Injuries and Risk Factors Study (GBD), in which it is argued that the lack of a safety limit for alcohol consumption because the apparent benefit obtained in the evolution of cardiovascular diseases is significantly reduced by other risks for health, mainly from cancer [31]. Moreover, a strong correlation was demonstrated between chronic alcohol consumption, progressive liver damage, and increased mortality in these patients [32]. As “complete abstinence” is difficult to accept by a majority of patients, possibly due to sociocultural reasons, European guidelines recommend a reduction in consumption to ≤2 drinks/day for women and ≤3 drinks/day for men, which is equivalent to 20 g, respectively, or 30 g of alcohol [33]. Although there are differences between the opinions of specialists regarding the involvement of the factors responsible for the generation of liver diseases, the substantial contribution attributed to the amount and duration of alcohol consumption is unanimously accepted [34].

Cumulative data from different epidemiological studies support the hypothesis of a direct dependence of the risk of liver disease on the amount of alcohol consumed [35]. French researchers were the first to establish that a minimum dose of 80 g of alcohol/day favors the appearance of cirrhosis [27]. Instead, Simpson et al. suggested that a reduced threshold of daily alcohol consumption (30 g in women and 50 g in men) could be considered, with a duration of at least 5 years being necessary to induce the onset of clinically significant liver [36].

Recently, Schwarzinger et al. demonstrated that there is a strong causal relationship between alcohol consumption and the onset of adverse effects when the weekly quantity of ethanol ingested exceeds 100 g [37]. Moreover, an excessive intake of alcohol in a repetitive manner (minimum 60 g/episode in men and 40 g/episode in women) causes the generation of toxic metabolites that trigger inflammatory cascades that activate oxidative stress and lipid peroxidation [38]. Several researchers have supported the idea of early detection of advanced fibrosis caused by alcohol as part of a process to improve long-term survival in association with supportive therapy to reduce excessive consumption and inclusion in screening programs to detect portal hypertension and hepatocellular carcinoma [39,40].

Although liver biopsy is considered the gold standard for evaluating fibrosis, its use is limited due to the potential risk of serious complications occurring in approximately 1–3% of cases, with a mortality rate of 1 in 10,000 cases [41]. However, TE is a method with reproducible results and which has the advantage of non-invasively investigating a large area of the liver parenchyma [40,41,42].

A meta-analysis by Pavlov et al. reported a 92% sensitivity for detecting advanced fibrosis in patients with alcohol consumption using TE with a cut-off around 9.5 kPa, with values between 8 and 10.5 kPa [43]. Another recent meta-analysis was conducted on 5.648 patients, 946 of whom were diagnosed with ALD, and reported a 94% sensitivity for advanced fibrosis using 8 kPa as a cut-off [44]. Therefore, the last guideline on noninvasive assessment of liver disease severity recommends ruling out advanced fibrosis in patients with alcohol consumption with a result below 8–10 kPa using TE [20].

Our results support the idea that there are major differences regarding the impact of alcohol on health depending on the type of preferred drink and the particularities of consumption (moderate versus excessive), which are objectified by the abnormal changes of specific biomarkers for inflammatory status and oxidative stress. Early detection of liver fibrosis through TE could prevent the development of complications and the evolution towards alcoholic liver disease and cirrhosis that have a strong socio-economic impact on patients and healthcare systems, an idea highlighted in a study conducted by Trifan et al. in which from over 1429 patients diagnosed for the first time with alcoholic liver cirrhosis during two and a half years, more than 20.8% deceased due to decompensation of the hepatic disease [45]. Improving compliance and applying monitoring strategies, specific therapy, and psychological counseling could contribute to a better prognosis for these patients. Moreover, the findings of this study could be the basis for the development of public health programs in order to promote the reduction of alcohol consumption.

However, the study has several limitations. The information was obtained through self-reports from the investigated subjects, which was a risk of communicating incorrect data, possibly for psychological, social, and educational reasons that could lead to an underestimation of the relationship between the way of consumption and the effects on health. Moreover, periodic follow-up should be considered for all patients who admit alcohol consumption since we could not estimate the evolution of those with moderate consumption or the changes in drinking patterns. Since this evaluation was carried out in a single medical center and did not include a sample represented by people who declared themselves abstinent, the possibility of generalizing the results was greatly reduced. Another limitation of the study is the impossibility of confirming or ruling out the overlap of non-alcoholic steatohepatitis (NASH) and ALD since we did not perform a liver biopsy. Those presenting risk factors for NASH were scheduled for further evaluation.

## 5. Conclusions

Screening using TE for patients who admit alcohol consumption represents an approach that allows an early diagnosis of advanced liver fibrosis in an asymptomatic population, being useful for the development of a risk stratification scheme. Moreover, it could be the basis of a personalized management algorithm for the implementation of early interventions that might prevent the possibility of alcoholic liver disease development and complications of cirrhosis.

## Figures and Tables

**Table 1:** Main characteristics of the study population according to the stage of liver fibrosis.

Variables		Overall Cohort	Participants with Fibrosis ≤F2	Participants with Fibrosis ≥F3
Patients ^a^		689	556 (80.7)	133 (19.3)
Age (years) ^b^		48 (40 to 61)	46 (38 to 56)	60 (48.5 to 65)
Gender ^a^	male	405 (58.8)	308 (55.4)	97 (72.9)
	female	284 (41.2)	248 (44.6)	36 (27.1)
Age groups ^a^	≤40 years	174 (25.3)	161 (29.0)	13 (9.8)
	>40 and ≤55 years	282 (40.9)	245 (44.1)	37 (27.8)
	≥55 years	233 (33.8)	150 (26.9)	83 (62.4)
Hypertension ^a^		258 (37.4)	213 (38.3)	45 (33.8)
T2DM ^a^		103 (14.9)	80 (14.4)	23 (17.3)
BMI (kg/m^2^) ^b^		22.7 (20.3 to 25.4)	22.79 (20.5 to 25.74)	22.48 (20.23 to 25.03)
Waist circumference (cm^2^) ^b^		86 (64 to 97)	88 (67 to 103)	83 (59 to 87)
Pattern of alcohol consumption ^a^	moderate	557 (80.4)	528 (94.7)	29 (21.8)
	excessive	132 (19.6)	28 (5.3)	104 (78.2)

Data expressed as ^a^ absolute (%) or ^b^ median [IQR], BMI-body mass index, T2DM-type 2 diabetes mellitus.

**Table 2:** Biologic parameters according to alcohol consumption.

Variables ^a^	Alcohol Consumption			
	Moderate n = 557 Patients		Excessive n = 132 Patients	
	Median	IQR	Median	IQR
Platelet count, 10^3^/µL	220	185 to 259	192	144 to 220
Fibrinogen, mg/dL	322	284 to 370	329	291 to 327
INR	1.08	1.01 to 1.15	1.11	1.02 to 1.20
CRP, mg/dL	0.38	0.3 to 0.42	0.45	0.36 to 0.54
Ferritin level, ng/L	129	94 to 189	131	101 to 184
Fasting plasma glucose, mg/dL	82	74 to 93	87.12	77 to 98.1
Urea, mg/dL	41	32 to 47	40	30 to 47
Creatinine, mg/dL	0.84	0.76 to 0.94	0.86	0.79 to 0.97
ALT, IU/L	42.56	31.3 to 54.2	58.25	33 to 72
AST, IU/L	37.10	27 to 52	51.27	37.25 to 61
GGT, IU/L	61	42 to 83	82.3	61.25 to 106.25
ALP, IU/L	87	71 to 112	97.5	72 to 133.5
Total bilirubin, mg/dL	0.73	0.57 to 0.97	0.91	0.67 to 1.20
Total cholesterol, mg/dL	210	190 to 237	214	174 to 245
Triglycerides, mg/dL	150.34	120 to 186	162	129 to 189
Albumin, g/dL	4.56	4.34 to 4.67	3	2.80 to 4.49
LDL-c, mg/dL	129	102 to 160	132	88 to 157
HDL-c, mg/dL	43	35.5 to 51	42	35 to 53
Uric acid, mg/dL	5.2	4.2 to 6.8	5.6	4.2 to 6.8
Alpha-fetoprotein, ng/mL	3.9	2.85 to 5.17	4.16	3.10 to 6.11

Data expressed as ^a^ median and [IQR]-interquartile range, CRP: C-reactive protein, ALT: alanine aminotransferase, AST: aspartate aminotransferase, GGT: gamma-glutamyl transferase, ALP: alkaline phosphatase, LDL-c: low-density lipoprotein cholesterol, HDL-c: high-density lipoprotein cholesterol.

**Table 3:** Biological modification according to gender and preferred beverage.

Advanced Fibrosis ≥F3	Male, n = 97 Patients				Female, n = 34 Patients		
	Wine n = 27 Patients	Beer n = 23 Patients	Distilled Spirits n = 47 Patients	*p*	Wine n = 17 Patients	Distilled Spirits n = 17 Patients	*p*
	Mean ± SD	Mean ± SD	Mean ± SD		Mean ± SD	Mean ± SD	
Platelet count, 10^3^/µL	187.15 ± 41.44	193.22 ± 33.98	177.63 ± 58.11	0.128	190.88 ± 49.79	174.29 ± 56.75	0.174
Fibrinogen, mg/dL	339.04 ± 47.94	325.78 ± 59.97	312.55 ± 60.93	0.165	311.29 ± 55.84	347.10 ± 64.02	0.076
INR	1.11 ± 0.24	1.12 ± 0.16	1.11 ± 0.17	0.788	1.14 ± 0.23	1.04 ± 0.29	0.468
CRP, mg/dL	0.30 ± 0.0 9	0.33 ± 0.11	0.59 ± 0.31	0.038	0.28 ± 0.10	0.68 ± 0.42	0.031
Ferritin level, ng/L	158.37 ± 98.60	155.53 ± 58.05	167.72 ± 69.64	0.534	109.76 ± 21.54	112.54 ± 38.84	0.917
Fasting plasma glucose, mg/dL	85.23 ± 24.45	88.22 ± 22.89	91.38 ± 28.98	0.709	86.17 ± 26.33	89.29 ± 27.17	0.798
Urea, mg/dL	35.44 ± 9.42	40.35 ± 10.48	41.15 ± 12.66	0.370	38.04 ± 11.58	40.06 ± 10.98	0.959
Creatinine, mg/dL	0.86 ± 0.15	0.94 ± 0.16	0.89 ± 0.19	0.320	0.91 ± 0.13	0.85 ± 0.10	0.214
AST, IU/L	36.88 ± 14.48	42.08 ± 13.60	62.13 ± 20.78	0.016	50.88 ± 21.59	54.94 ± 24.59	0.417
ALT, IU/L	40.40 ± 10.46	45.22 ± 14.24	71.32 ± 26.72	0.035	59.12 ± 17.71	60.47 ± 27.17	0.678
GGT, IU/L	57.85 ± 30.06	67.69 ± 32.90	86.29 ± 39.91	0.114	68.24 ± 27.94	82.12 ± 38.22	0.094
ALP, IU/L	90.22 ± 43.66	101.30 ± 34.35	105.55 ± 38.98	0.219	93.94 ± 42.67	102.06 ± 37.72	0.324
Total bilirubin, mg/dL	0.86 ± 0.44	0.88 ± 0.51	0.92 ± 0.55	0.604	0.91 ± 0.56	1.05 ± 0.58	0.241
Total cholesterol, mg/dL	164.15 ± 40.88	194.22 ± 45.61	215.42 ± 48.15	0.037	188.82 ± 41.13	264.59 ± 57.85	0.028
Triglycerides, mg/dL	171.70 ± 56.33	165.91 ± 41.82	207.13 ± 60.17	0.023	182.47 ± 54.71	191.24 ± 56.54	0.214
Albumin, g/dL	2.92 ± 0.48	3.15 ± 0.65	2.97 ± 0.57	0.441	3.67 ± 0.61	3.25 ± 0.58	0.543
LDL-c, mg/dL	87.37 ± 23.37	107.65 ± 28.64	135.47 ± 34.32	0.013	96.47 ± 27.49	143.53 ± 29.47	0.037
HDL-c, mg/dL	38.59 ± 10.69	41.04 ± 10.99	37.62 ± 10.51	0.352	42.47 ± 11.69	38.88 ± 10.31	0.429
Uric acid, mg/dL	5.32 ± 1.33	5.58 ± 1.43	5.48 ± 1.30	0.685	4.87 ± 1.15	4.83 ± 1.08	0.457
Alpha-fetoprotein, ng/mL	4.75 ± 1.42	4.98 ± 1.62	4.86 ± 1.57	0.491	4.81 ± 1.43	4.72 ± 1.28	0.577

SD: standard deviation, CRP: C-reactive protein, ALT: alanine aminotransferase, AST: aspartate aminotransferase, GGT: gamma-glutamyl transferase, ALP: alkaline phosphatase, LDL-c: low-density lipoprotein cholesterol, HDL-c: high-density lipoprotein cholesterol.

**Table 4:** Risk for advanced fibrosis according to age and preferred beverage.

Age	Beverage	Moderate Alcohol Consumption	Excessive Alcohol Consumption
		OR (95% CI)	OR (95% CI)
≤40 years	Wine	1.2 (0.28–5.22)	0.67 (0.10–4.35)
	Beer	0.38 (0.07–1.95)	0.42 (0.07–2.65)
	Distilled spirits	2.40 (0.54–10.61)	3.50 (0.59–20.69)
>40 and ≤55 years	Wine	1.04 (0.26–4.13)	0.40 (0.12–1.34)
	Beer	1.18 (0.40–3.48)	1.19 (0.36–3.99)
	Distilled spirits	1.24 (0.38–4.14)	1.56 (0.53–4.63)
>55 years	Wine	0.71 (0.18–2.80)	1.63 (0.48–5.54)
	Beer	0.57 (0.14–2.26)	0.82 (0.021–1.23)
	Distilled spirits	3.62 (1.13–11.64)	3.73 (1.51–14.63)

**Table 5:** Predictive factors for advanced fibrosis according to gender and age in univariate analysis.

Biologic Parameters	Male		Female		≤40 Years		>40 Years and ≤55 Years		>55 Years	
	*β*	*p*	*β*	*p*	*β*	*p*	*β*	*p*	*β*	*p*
Platelet count, 10^3^/µL	−0.304	0.001	−0.387	0.001	−0.235	0.001	−0.237	0.001	−0.469	0.001
ALT, IU/L	0.167	0.001	0.240	0.001	-	-	0.124	0.037	0.278	0.001
AST, IU/L	0.145	0.003	0.212	0.001	-	-	0.136	0.022	0.290	0.001
GGT, IU/L	-	-	0.458	0.001	-	-	-	-	-	-
Ferritin level, ng/L	-	-	−0.134	0.024	-	-	-	-	0.159	0.015
CRP, mg/dL	-	-	−0.217	0.001	-	-	-	-	-	-
Total bilirubin, mg/dL	0.257	0.001	0.232	0.001	0.233	0.020	0.257	0.001	0.314	0.001
Total cholesterol, mg/dL	-	-	−0.559	0.001	-	-	−0.383	0.021	−0.456	0.001
Triglycerides, mg/dL	0.343	0.036	0.119	0.045	-	-	-	-	-	-
Albumin, g/dL	−0.752	0.001	−0.450	0.001	−0.730	0.001	−0.640	0.001	−0.570	0.001
LDL-c, mg/dL	-	-	−0.380	0.001	-	-	-	-	0.384	0.001

CRP: C-reactive protein, ALT: alanine aminotransferase, AST: aspartate aminotransferase, GGT: gamma-glutamyl transferase, LDL-c: low-density lipoprotein cholesterol.

## Data Availability

The data presented in this study are available on request from the corresponding author. The data are not publicly available because they are the property of the Institute of Gastroenterology and Hepatology, Iasi, Romania.
